# Development and Promotion of Concrete Strength at Initial 24 Hours

**DOI:** 10.3390/ma16124452

**Published:** 2023-06-18

**Authors:** Chuanhe Fan, Jueshi Qian, Huaqiang Sun, Yingru Fan

**Affiliations:** 1 College of Materials Science and Engineering, Chongqing University, Chongqing 400044, China; 2 School of Advanced Manufacturing, Fuzhou University, Quanzhou 362200, China

**Keywords:** compressive strength, earlier age, concrete, temperature, calcium sulfoaluminate cement, silica fume, long-term performance

## Abstract

Knowing and promoting the strength development of concrete at an earlier age is essential for accelerating formwork circulation of the on-site construction and precast product manufacture. The strength development rate at earlier ages of less than the initial 24 h was investigated. The effect of measures of adding silica fume, calcium sulfoaluminate cement, and early strength agent on the strength development of earlier concrete at ambient temperatures of 10, 15, 20, 25, and 30 °C was studied. The microstructure and long-term properties were further tested. It is shown that the strength increases exponentially first and then logarithmically, different from what is commonly recognized. Increasing cement content exhibited a certain effect only above 25 °C. When the cement content increased from 420 to 460 kg/m^3^, the strength only increased from 6.2 to 6.7 MPa after 12 h at 25 °C. The early strength agent could increase the strength significantly, the strength could be increased from 6.4 to 10.8 MPa after 20 h at 10 °C and from 7.2 to 20.6 MPa after 14 h at 20 °C. All measures for promoting earlier strength did not have an evident negative effect. The results could be potentially referred for the formwork removal at a suitable moment.

## 1. Introduction

Concrete is the most widely used construction material around the world [1,2], commonly made by mixing cement with aggregate, water, and admixture [3,4,5,6,7]. Compared with other materials, concrete shows a lot of merits, such as rich sources of raw materials, low cost, simple preparation process, and excellent mechanical properties [8]. As a structural material, compressive strength is the most important index to characterize the performance of concrete [9,10]. With the advancement of civil engineering technology and the requirement for low-carbon emissions, it has become a common demand to improve construction efficiency reasonably. The early age strength of concrete has attracted more and more attention. The term early age only covers the initial time in the total life of the concrete. The time before 7 d is usually regarded as the early age [11,12,13]. In general, the development of compressive strength abides by a logarithmic law in all ages including the early age [14,15].

When compressive strength at an early age does not meet a practical requirement, some strength promotion methods need to be used [16,17,18]. A commonly used method is to add some materials to promote early age compressive strength, which can avoid or reduce additional maintenance measures simultaneously. Of all the materials, early strength agent is the most widely used, and very effective. For example, C-S-H seed and sodium sulfate exhibit a synergistic enhancement effect on promoting early age strength, and the combination of them as an early strength agent can promote compressive strength from 4 MPa to 15 MPa at 1 d [19]. The composite early strength agent, prepared with calcium bromide (CaBr_2_), lithium bromide (LiBr), and triisopropanolamine (TIPA), can increase the compressive strength from 2.4 MPa to 9.4 MPa at 1 d, and from 26.8 MPa to 47.7 MPa after 3 d [20]. Furthermore, calcium sulfoaluminate cement [21,22,23,24], calcium aluminate cement [25,26], nanoparticles [27,28,29,30], crystal seeds [31,32,33,34], graphene oxide [35,36,37,38], and so on can also be used as these materials. Except for the early strength materials mentioned above, thermal insulation [14,39] and thermal curing [40,41,42,43] also have been used to accelerate the development of strength.

At present, studies on early age strength are mainly focused on 3 to 7 d, with little attention paid to strength at earlier ages of less than 1 d. However, the strength before 3 d, especially within 1 d, is becoming more and more important for modern civil engineering. For cast-in-place concrete, the earlier strength determines the time to remove the formwork and the timing for the next construction process [44]. For precast concrete products, the earlier strength determines the circulation rate of the formwork and affects production efficiency directly. If the characteristics and development rate of strength during this period are misunderstood, improper operations may be taken then, resulting in a negative effect on the consequent strength development and the durability of the structure [45,46]. Unfortunately, there is no clear research result on the characteristics and development rate of concrete strength within 1 d. In addition, there is little research about the effect of the early strength materials on strength development at earlier ages of less than 24 h.

The present study aims to investigate the compressive strength development rate and the effect of ambient temperature and early strength materials including silica fume, calcium sulfoaluminate cement, and early strength agent on strength development within 1 d. Long-term experiments on strength up to 3 years and chloride ion permeability were carried out, to clarify the effect of these technologies for promoting earlier strength on the long-term performance of concrete. Based on the strength development rate at an earlier age, it is expected to evaluate the effectiveness of different promotion technologies by measuring the impact of these measures on earlier age strength and long-term performance.

## 2. Materials and Methods

### 2.1. Materials

Concrete was prepared with cement, manufactured sand, crushed rock, water, and superplasticizer, as a blank group. Ordinary Portland cement was PO42.5R with an apparent density of 3015 kg/m^3^, marked as OPC. Two types of crushed rocks with different particle sizes were mixed in a mass ratio of 4:6, as coarse aggregate, and the particle sizes are 5~10 mm and 10~20 mm, respectively, the apparent density of both types of crushed rock is 2670 kg/m^3^. The OPC, sand, and crushed rock are all from Chongqing, China. A polycarboxylate water-reducing agent, marked as PCA, was added to improve the workability.

Silica fume, calcium sulfoaluminate cement, and early strength agent were also used for accelerating strength development. The specific surface area of silica fume (SF) is 24,500 m^2^/kg and the average particle size is about 0.15 μm. The calcium sulfoaluminate cement is from Henan, China, and the type is R-SAC42.5, marked as CSA. The early strength agent is a milky white liquid, with the core components of C-S-H-PCE nanocomposites [47], and the dosage was 5% of the mass of cement, marked as CCP. The chemical compositions of OPC, SF, and CSA are shown in Table 1.

### 2.2. Concrete Mixture and Sample Preparation

The mixture proportions of blank group concrete are shown in Table 2. The sand ratio was 39%, the water-to-binder ratio was 0.30, and the dosage of PCA was 0.6% of the mass of cementitious material. When SF and CSA were added to the concrete, the same mass of OPC as SF or CSA would be replaced. Similarly, when CCP was used, the same amount of water as CCP would be replaced.

All raw materials for preparing concrete, molds, mixer, and other tools which touch concrete mixture during mixing and casting were kept in the environment with a designed constant temperature. After mixing fully, cast the concrete mixture into concrete molds with the size of 100 mm × 100 mm × 100 mm, then vibrated for 30 s on the vibration table. After casting, the samples/specimens were cured in a box with the designed constant temperature and relative humidity of 90%. When testing the earlier strength, removed the molds 5 min before the test. When testing the long-term strength, the demolding time was 1 d, and then placed in the constant temperature and humidity box again for curing. After 28 d, the specimens were taken out and placed in an indoor environment.

### 2.3. Test Methods

The compressive strength test was carried out according to GB/T 50081-2019 [48]. The temperature variation of concrete was recorded using the temperature recorder (Elitech, RC-4, Shanghai, China) with a temperature sensor embedded in the concrete core. The chloride migration coefficient of concrete at 28 d was performed according to GB/T 50082-2009 [49]. The mineral compositions of the hydration product were determined by X-ray diffraction (XRD) (SpectrisPte. Ltd., Singapore) with a scanning rate of 2°/min. SEM analysis was conducted to observe the morphology of the hydration product. Before the test, the specimen was sprayed with gold.

## 3. Results and Discussion

### 3.1. Strength Development and Temperature Variation of Concrete within 1 d

**Figure 1:**
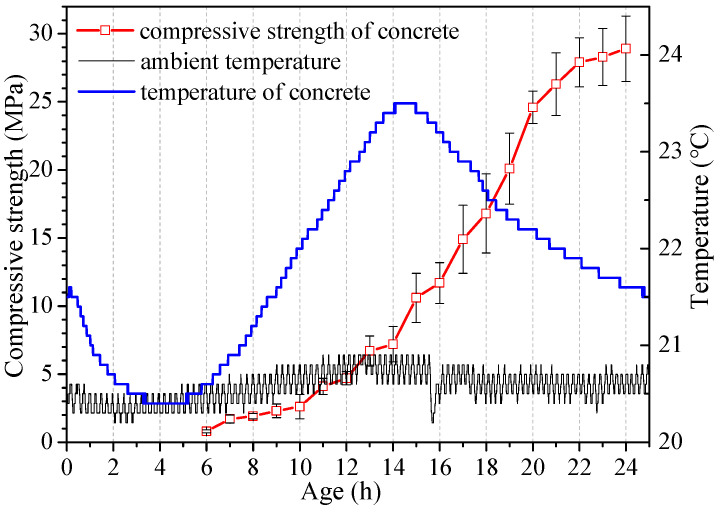
Strength development rate and temperature variation of concrete at 20 °C.

Figure 1 shows the compressive strength development rate and corresponding temperature variation within 1 d at an ambient temperature of about 20 °C. In order to avoid the influence of supplementary cementitious material, only cement was used to prepare concrete. The mix proportions are shown in Table 1 and the ambient temperature was 20 °C. The strength was tested from 6 h to 24 h with an interval of 1 h. Meanwhile, the ambient temperature and temperature at the core of the specimen within 1 d were recorded continuously.

It can be seen from Figure 1 that the compressive strength always increases with age from 6 h to 24 h, but the strength development rate is different from that commonly recognized. It is generally believed that the strength increases logarithmically with age, that is, the strength increases rapidly in the early age and tends to slow down in the later age. However, the strength of concrete does not directly exhibit a rapidly increasing pattern within the age of 1 d. There is a latent stage of strength development first, following a rapid increase stage, and then a stage of gradual slow down. The strength increases very slowly within 14 h, rapidly at 14~20 h, and gradually slows down at 20~24 h. It is concluded that the compressive strength of concrete within 1 d increases exponentially at first and then logarithmically. Previous studies believed that compressive strength increases logarithmically but ignored that there is a stage of exponential increase in concrete strength.

It also can be seen from Figure 1 that there is a clear relationship between the strength development and the temperature of the concrete. The temperature of concrete increases rapidly at once when concrete was cast into the mold, but then decreases until the first 4 h, corresponding to the initial fast reaction period and induction period of cement [50]. The corresponding strength development at the first 6 h is extremely low. Then the temperature rises at 6~14 h, indicating that the hydration rate is gradually accelerating. Additionally, the strength development begins to accelerate at 8 h, exhibiting exponential increase. Concrete has the highest temperature at about 14 h due to the intensive heat release of cement hydration. In addition, the corresponding strength increases more rapidly from 14 h. The temperature gradually decreases after 14 h but maintains at a relatively high level during 14~20 h, therefore the strength still increases rapidly during this period. The hydration heat gradually decreases to a low level after 20 h, and the strength development rate decreases correspondingly.

The temperature rise of concrete is related to the hydration of cement. It is well known that cement has four stages of hydration with different characteristics. It is suggested that the strength development of concrete can be predicted by monitoring the temperature variation of concrete. The temperature curve can reflect several characteristics of the strength development rate. (1) The temperature begins to increase corresponding to the start of the strength increase (6 h). (2) The temperature reaches its peak corresponds to rapid strength development. (3) The period of several hours after the temperature peak is crucial, as it is a rapid increase stage of concrete strength. It is suggested that measures that promote the temperature rise of concrete would accelerate the strength development of earlier concrete.

### 3.2. Effect of Factors on Strength Development

Figure 1 shows that the strength of concrete was relatively low within 1 d, especially at the earlier age within 8 h or 12 h. Sometimes, higher strength is assured to meet the requirement of formwork circulation and timing of thermal curing. There are a lot of measures promoting the early strength of concrete, but fewer experimental results related to the earlier age within 24 h. The early strength development of concrete has a close relationship with the temperature inside the concrete. Low ambient temperature will reduce the temperature rise of concrete. Therefore, when the effect of measures on promoting the earlier strength of concrete was investigated, ambient temperatures at 10 °C, 15 °C, 20 °C, 25 °C, and 30 °C were set. In order to reduce the experiment quantity, the four earlier ages of less than 20 h were selected.

(1) Increasing content of Portland cement

**Figure 2:**
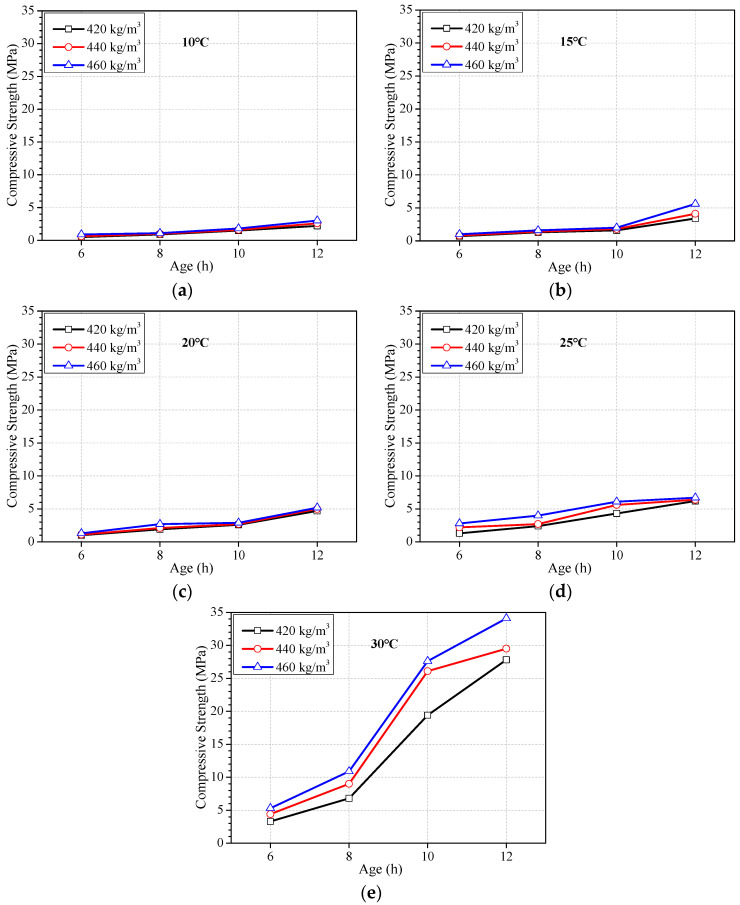
Strength development of concrete with different cement contents and constant water-to-cement ratio of 0.30. Ambient temperature at (**a**) 10 °C, (**b**) 15 °C, (**c**) 20 °C, (**d**) 25 °C, and (**e**) 30 °C.

Table 3 lists the mixture proportion of concrete when cement content was 420 kg/m^3^, 440 kg/m^3^, and 460 kg/m^3^, respectively. The ratio of water to cement was kept at 0.30 constantly. Other components of concrete were adjusted correspondingly. Figure 2 shows that strength at 6 h, 8 h, 10 h, and 12 h varied with cement content when the ambient temperature was 10 °C, 15 °C, 20 °C, 25 °C, and 30 °C, respectively.

It can be seen from Figure 2 that the strength development does not exhibit an evident increase with increasing cement content when the ambient temperature is less than 25 °C. When the temperature is above 25 °C, the effect of cement content on the strength development starts to be evident. However, it is not effective to promote the strength at 6~12 h by increasing cement content. Although the strength increases significantly with the increase in cement content when the ambient temperature is 30 °C, the strength of concrete with 420 kg/m^3^ is relatively too high. Compared with the strength development in Figure 1, the ambient temperature is a crucial factor, or it is not necessary to adopt measures promoting strength when the ambient temperature is high enough, such as higher than 30 °C in this work. In a word, the effect of increasing cement content on promoting the earlier strength of concrete is very limited.

(2) Addition of SF

**Figure 3:**
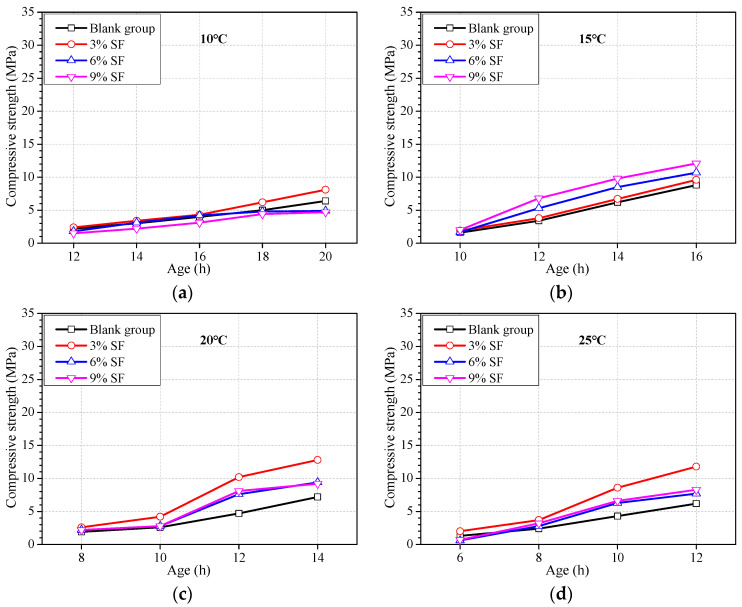

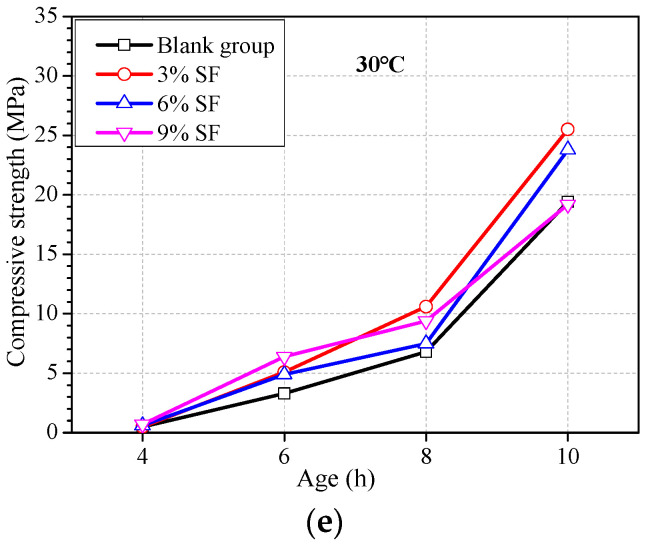
Strength development of concrete with the addition of SF at different ambient temperatures. Ambient temperature at (**a**) 10 °C, (**b**) 15 °C, (**c**) 20 °C, (**d**) 25 °C, and (**e**) 30 °C.

The addition of SF can usually promote the strength development of concrete significantly. Adding SF has become a common way of preparing high-strength concrete. The previous literature [14,51] has shown that SF can improve early strength, but it is not verified whether the SF can improve the earlier strength within 1 d. Figure 3 shows the effect of SF on the strength development of concrete at ages of less than 20 h. The mixture proportion was basically the same as that in Table 1, and the content of SF was 3%, 6%, and 9%, just replacing an equal quantity of cement.

It can be seen from Figure 3 that the SF, to some extent, can promote the strength of concrete except the ambient temperature is 10 °C. However, the effect of SF on the strength enhancement varies with the content of SF, the ambient temperature, and age. When the temperature is at 20 °C or above, SF of 3% instead of 9% can achieve the highest enhancement of early strength at ages of less than 16 h. The extent of strength promotion increases with age. It is suggested that SF can be used to promote the earlier strength of concrete as long as the ambient temperature is above 15 °C, and there is a suitable content of SF for promoting the earlier strength of concrete at an age of less than 20 h.

(3) Addition of calcium sulfoaluminate cement (CSA)

**Figure 4:**
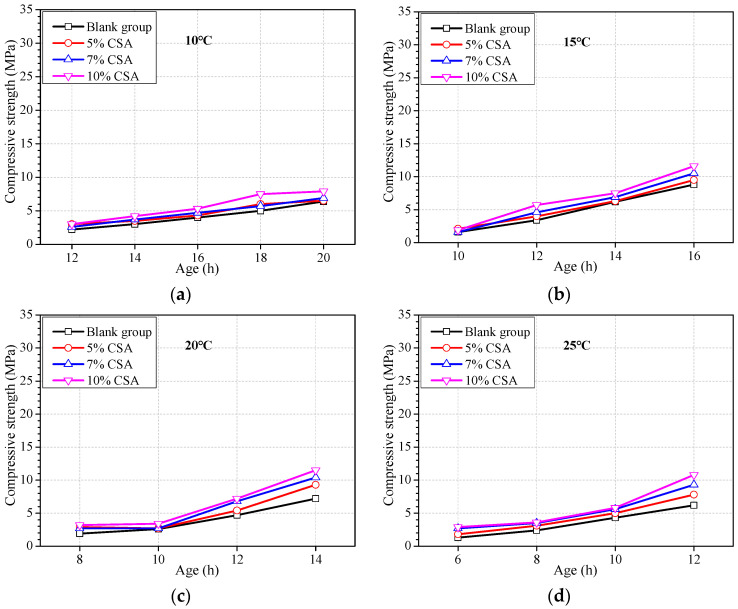

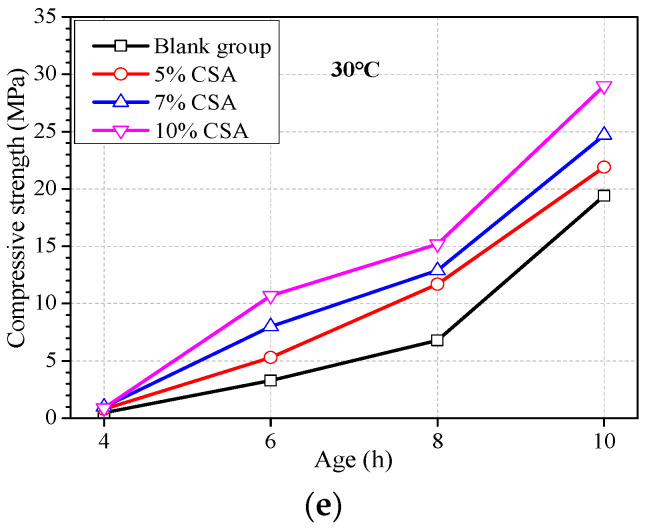
Strength development of concrete with the addition of CSA at different ambient temperatures. Ambient temperature at (**a**) 10 °C, (**b**) 15 °C, (**c**) 20 °C, (**d**) 25 °C, and (**e**) 30 °C.

Concrete made from CSA can obtain higher early strength even in low ambient temperatures [52,53]. Previous works have confirmed the positive effect of the addition of CSA in OPC on early strength development [54,55]. However, the effect of the addition of CSA in OPC concrete on the concrete strength at a much earlier age and relatively lower ambient temperature is not clear. Figure 4 shows the strength development of concrete with 5%, 7%, and 10% CSA, replacing the same quantity of OPC, at ages of less than 20 h and at ambient temperatures of 10 °C, 15 °C, 20 °C, 25 °C, and 30 °C.

It can be seen from Figure 4 that CSA has a certain effect on promoting earlier strength within 1 d regardless of ambient temperatures, but no significant effect until the temperature is above 25 °C. It can be concluded that the addition of CSA in OPC concrete can be used as a way to promote earlier strength only to a certain extent. At the ambient temperature of 30 °C, the strength promotion at an age of 6 h increased more than doubled with CSA of 7%. It indicates that the addition of CSA is an effective way to promote earlier strength when the ambient temperature is relatively high.

(4) Addition of early strength agent

**Figure 5:**
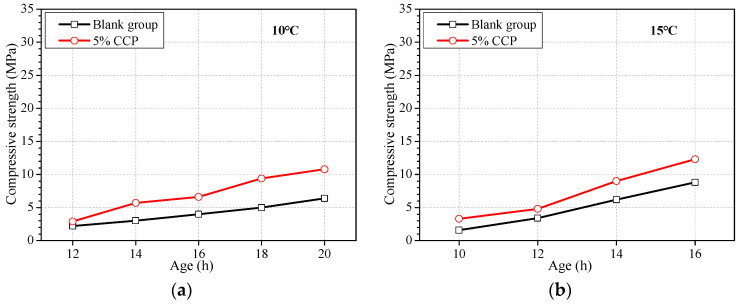

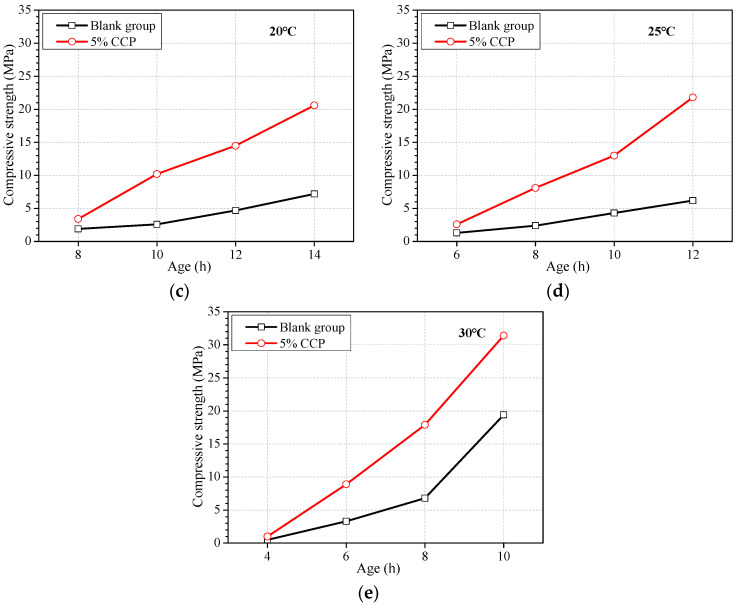
Strength development of concrete with addition of CCP at different ambient temperatures. Ambient temperature at (**a**) 10 °C, (**b**) 15 °C, (**c**) 20 °C, (**d**) 25 °C, and (**e**) 30 °C.

Figure 5 shows the effect of an early strength agent on the earlier strength. The early strength agent CCP is a kind of crystal seed containing C-S-H [47]. It can be seen from Figure 5 that the agent CCP can promote significantly the earlier strength of concrete at ages of less than 1 d even if at the ambient temperature of 10 °C. The agent CCP of 5% increases the strength by 65% at the age of 16 h, and by 69% at the age of 20 h when the ambient temperature is 10 °C. Similarly, the agent CCP is more effective in promoting the earlier strength of concrete at ages of less than 20 h when the ambient temperature is more than 15 °C.

(5) Discussion

Among the four ways above, increasing cement content is difficult to promote effectively the earlier strength of concrete, especially at a lower ambient temperature. This depends on the characteristic of hydration heat release because cement needs enough time to accumulate a certain amount of heat. However, additions of SF, CSA, and early strength agent CCP would provide extra driving heat to accelerate the hydration of cement. Compared with the other ways above, the addition of an early strength agent is a more effective way of promoting the earlier strength.

It is worth noting that most experimental results of strength at ages of less than 24 h exhibit an exponential increase within a certain period varied with the ambient temperature.

### 3.3. Microanalysis

The experimental results above show that all measures for promoting the earlier strength could to some extent enhance the strength development of concrete at ages of less than 24 h. The early strength agent CCP exhibited the most promotion efficiency on the strength at an earlier age even at an ambient temperature of 10 °C. The promotion mechanism of CCP on earlier strength was analyzed with XRD and SEM at a micro level. All samples that hydrated to the specified age were crushed into small pieces and then immersed in ethanol to terminate hydration. To prevent the very quick hydration rate of cement at an early age, ethanol was exchanged every 5 h, and the total soaking time was 24 h. After that, the sample was dried in a vacuum drying oven for 3 d. In the preparation of XRD test samples, the hydrating cement paste from the dried concrete was separated and sifted through 250 mesh. When observing the microscopic morphology with SEM, tests were directly carried out on the crushed concrete specimens.

The concrete samples with and without the early strength agent at the ambient temperatures of 10 °C and 30 °C were investigated. The concrete sample without the early strength agent hydrated for 18 h at 10 °C was marked as the 10 °C-18 h-blank group, and the sample with early strength agent was marked as 10 °C-18 h-CCP, similarly for those at 30 °C.

(1) XRD

**Figure 6:**
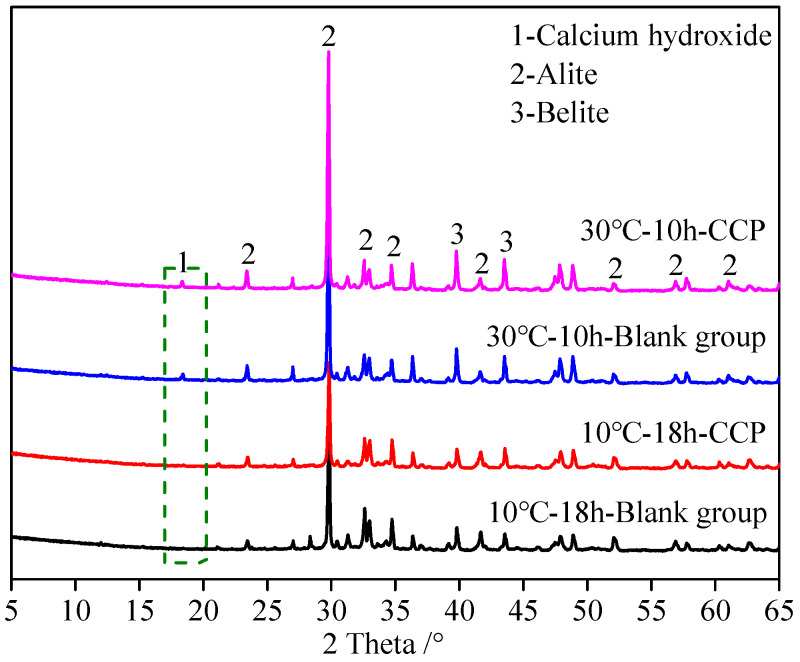
XRD patterns of concrete with and without early strength agent. Green dashed box: diffraction peak position of hydration product.

Figure 6 shows the effects of the early strength agent and the ambient temperature on the mineral compositions of hydration products at ages of 10 h and 18 h. No evident calcium hydroxide is observed from the XRD patterns at the ambient temperature of 10 °C even at the age of 18 h, regardless of whether the early strength agent was added or not. The strength of concrete with CCP increases by 4.4 MPa based on the strength of 5.0 MPa for concrete without CCP. When the ambient temperature is 30 °C, calcium hydroxide can be observed in the XRD pattern for concretes with and without CCP even at the earlier age of 10 h. Additionally, the strength of concrete with CCP increases by 12.0 MPa.

(2) SEM

**Figure 7:**
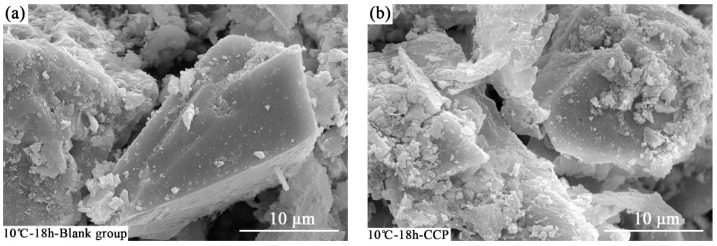

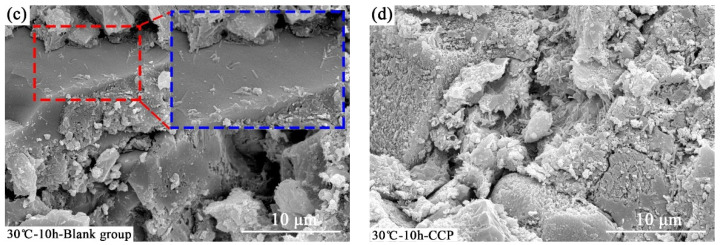
Microstructures of concrete with and without early strength admixture. (**a**) 10 °C-18 h-Blank group, (**b**) 10 °C-18 h-CCP, (**c**) 30 °C-10 h-Blank group; red dashed box: ettringite; blue dashed box: enlarged view of ettringite, (**d**) 30 °C-10 h-CCP.

Figure 7 shows that the microstructure of concretes with and without the early strength agent is different. There are more gel-like hydration products in the concrete with CCP compared with that without CCP. The hydration products may cover the cement particles. It also can be seen from Figure 7d that the gel-like hydration products include more needle-like products of ettringite at the temperature of 30 °C, being consistent with the XRD pattern in Figure 6. It is concluded that the early strength agent could enhance the formation of gel-like hydration products.

### 3.4. Long-Term Properties

Generally, when the early strength of concrete develops quickly, the long-term strength will slow down to some extent. Inappropriate early strength enhancement measures will even reduce the long-term strength and durability of concrete. Thus, it is necessary to investigate the long-term compressive strength and durability of concrete with the measures for promoting the earlier strength of concrete.

(1) Effect of measures for promoting earlier strength on the long-term strength of concrete

**Figure 8:**
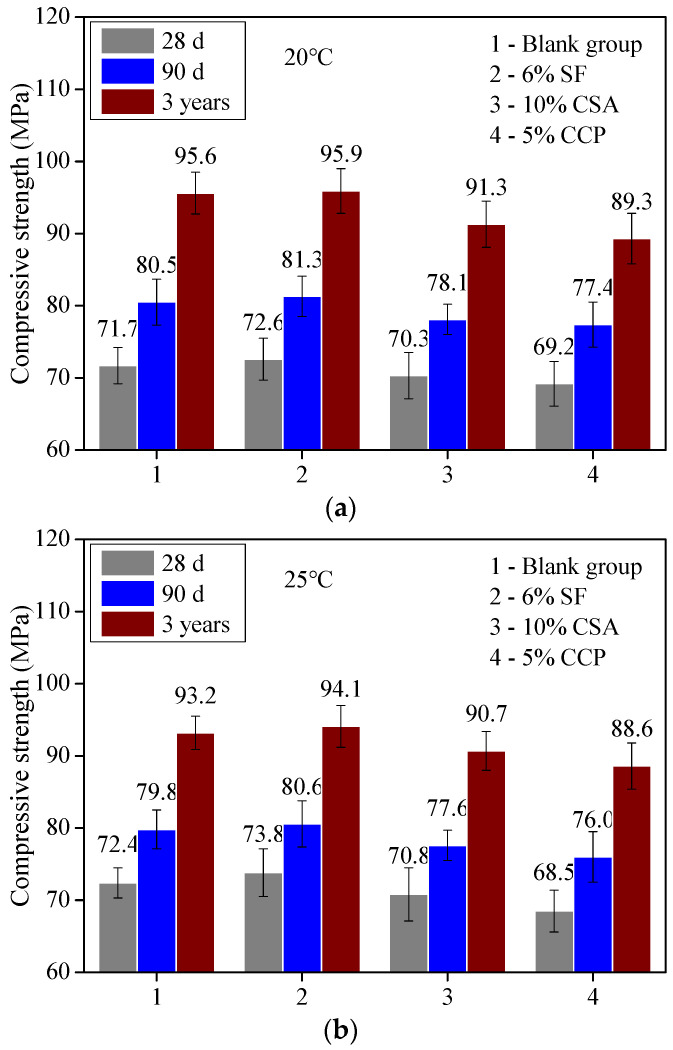

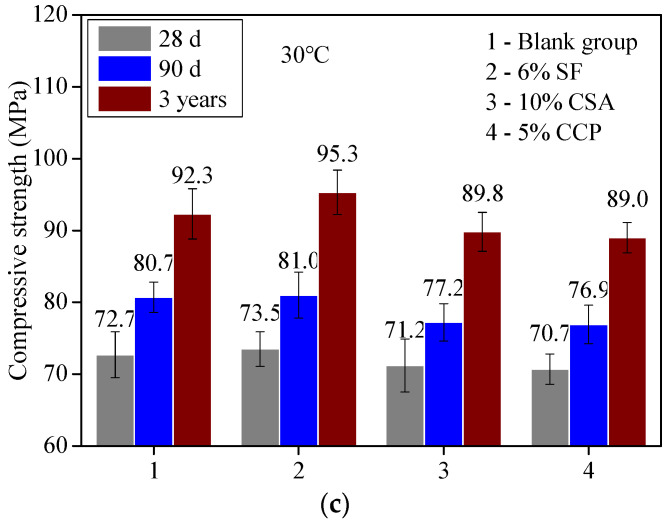
Long-term strengths of concrete with 6% SF, 10% CSA, and 5% CCP at ages 28 d, 90 d, and 3 years. Ambient temperature at (**a**) 20 °C, (**b**) 25 °C, (**c**) 30 °C.

Figure 8 shows the strength of concrete with 6% SF, 10% CSA, and 5% CCP at ages of 28 d, 90 d, and 3 years, at temperatures of 20 °C, 25 °C, and 30 °C, respectively.

As can be seen from Figure 8, the strength continued to increase significantly with age. Compared with the strength of concrete at 28 d, the strength at 90 d increases by more than 10%, and that at 3 years increases by about 30%. Among all measures, the addition of SF exhibits promotion efficiency for the earlier and long-term strength of concrete. The early strength agent CCP relatively exhibits more efficiency for earlier strength promotion but no effect on the strength at the ages of 90 d and 3 years, and even negative effects compared with concrete without any measure for promoting strength.

(2) Effect of measures for promoting earlier strength on chloride migration coefficient

**Figure 9:**
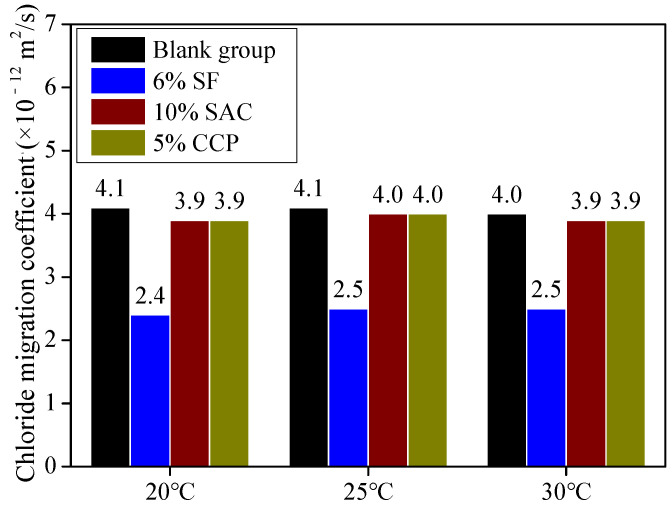
Chloride migration coefficients of concrete by means of measures for promoting earlier strength.

The resistance to chloride ion permeability can reflect the compactness of concrete and is a commonly used method to characterize its durability [56]. Figure 9 shows the effect of measures for promoting concrete strength at an earlier age on the chloride migration coefficient. It can be seen from Figure 9 that the addition of SF can significantly reduce the chloride migration coefficient, and other measures do not have an evident impact on the coefficient. It is concluded that all measures above have no negative influence and even a certain positive effect on the durability of concrete while promoting the strength of concrete at an earlier age.

## 4. Conclusions

This paper mainly investigated the development rate of concrete strength at an earlier age within 1 d under different ambient temperatures, especially at 10 °C, and the effect of some typical measures on strength promotion at an earlier age. The long-term strength and durability of concrete with the measures were tested at the same time. The following conclusions can be drawn:

(1) The strength of concrete at an earlier age within 1 d develops with a latent stage first, following an exponentially rapid rise stage, and then a logarithmically slow rise stage. The start time of an exponentially rapid rise corresponds to the highest temperature rise inside the concrete. The occurrence of the highest temperature within 1 d can be a key moment for the formwork removal of on-site concrete construction and precast concrete product manufacture.

(2) The ambient temperature dominates the earlier strength development of concrete. Increasing cement content is not effective for promoting earlier strength when the ambient temperature is lower than 20 °C.

(3) Measures including the addition of SF, CSA, and early strength agents can promote the earlier strength of concrete to different extents. The promotion effect is relatively evident under high ambient temperatures. The early strength agent CCP used in this work exhibits an evident effect even at a temperature of 10 °C.

(4) All measures for promoting earlier strength of concrete do not have evident negative effects on microstructure, long-term strength, and chloride migration coefficient of concrete, even the addition of SF exhibits positive effects. 

There is a good correlation between the early age strength and hydration heat of cement-based materials [57], but the hydration heat of concrete is not easily obtained in the actual environment. In this paper, it can be seen that the development of earlier age concrete strength is closely related to its temperature. Therefore, establishing a relationship between temperature and strength can not only reduce the workload of testing strength but also measure the effectiveness of different strength improvement technologies, which will be meaningful in future work.

## Figures and Tables

**Table 1:** Chemical compositions of OPC, SF, and CSA (wt%).

Sample	CaO	SiO_2_	Al_2_O_3_	Fe_2_O_3_	SO_3_	MgO	Others
OPC	54.79	26.16	6.93	3.36	2.92	2.91	2.93
SF	0.13	97.87	0.06	0.08	0.61	0.66	0.59
CSA	51.17	7.63	21.76	1.93	14.72	1.61	1.18

**Table 2:** Mixture proportions of blank group concrete per unit volume (kg/m^3^).

OPC	Sand	Fine Gravel	Coarse Gravel	Water	PCA
426	734	459	689	128	2.56

**Table 3:** Mixture proportions of concrete with different cement contents (kg/m^3^).

OPC	Sand	Fine Gravel	Coarse Gravel	Water	PCA
420	739	462	693	126	2.52
440	729	456	684	132	2.64
460	718	449	674	138	2.76

## Data Availability

Not applicable.
